# Omadacycline exhibits anti-inflammatory properties and improves survival in a murine model of post-influenza MRSA pneumonia

**DOI:** 10.1128/aac.00469-25

**Published:** 2025-08-04

**Authors:** Sumiko Gomi, Emily Price, Hailey Burgoyne, Sabrina Faozia, Eva Katahira, Eric McIndoo, Anyauba A. Nmaju, Kavita Sharma, Ali Aghazadeh-Habashi, Amy E. Bryant, Dennis L. Stevens, Jessica V. Pierce, Alisa W. Serio, Sarah E. Hobdey

**Affiliations:** 1 Idaho Veterans Research and Education Foundation, Boise, Idaho, USA; 2 Veterans Affairs Medical Center, Boise, Idaho, USA; 3 Boise State University 1791, Boise, Idaho, USA; 4 Idaho State University 212907, Meridian, Idaho, USA; 5 Idaho State University 6640, Pocatello, Idaho, USA; 6 University of Washington 7284, Seattle, Washington, USA; 7 Paratek Pharmaceuticals, Inc. 17822, King of Prussia, Pennsylvania, USA; Shionogi Inc., Florham Park, New Jersey, USA

**Keywords:** omadacycline, cytokines, immune response, mouse model, influenza, MRSA, pneumonia

## Abstract

Post-influenza *Staphylococcus aureus* pneumonia, particularly methicillin-resistant *S. aureus* (MRSA), remains a major cause of mortality, highlighting the urgent need for newer therapeutic options. Omadacycline is an aminomethylcycline antibiotic that has demonstrated efficacy against MRSA across many infection models, but its potential in post-influenza A virus (IAV)-MRSA pneumonia remains unexplored. Using a murine model of this infection, we evaluated the effects of omadacycline and a comparator antibiotic, linezolid, on survival, inflammation, bacterial load, toxin production, and lung histopathology. In survival studies, omadacycline matched the effectiveness of oral linezolid at clinically relevant doses. In addition, both antibiotics, particularly omadacycline, attenuated the production of multiple pro-inflammatory cytokines and reduced neutrophil infiltration in the lungs, independent of their effects on pulmonary microbial burden, suggesting an immunomodulatory mechanism of action. Panton-Valentine leukocidin (PVL) toxin production *in vitro* and *in vivo* was also assessed, and while the role of PVL in this murine model remains unclear, both agents reduced PVL production. These findings provide the first preclinical demonstration of *in vivo* efficacy for omadacycline against IAV-MRSA pneumonia and its ability to modulate the host immune response, thereby reducing the excessive inflammation that is linked to mortality in this disease state. Further investigation into the precise interplay between omadacycline and immunomodulation in this disease state is warranted.

## INTRODUCTION

Secondary bacterial pneumonia caused by methicillin-resistant *Staphylococcus aureus* (MRSA) is a leading cause of death in patients following influenza A virus (IAV) infection (1, 2). Despite the availability of MRSA-active antibiotics, mortality of post-influenza MRSA (IAV-MRSA) pneumonia remains high, and survivors require prolonged intensive care (3). IAV-MRSA pneumonia is characterized by widespread alveolar edema, neutrophilic infiltrate, hemorrhage, loss of lung structure, and necrosis (4). While a robust inflammatory response is required to kill bacteria and resolve infection, excessive and prolonged alveolar inflammation leads to severe lung injury and death (5–8). Compared with MRSA-only pneumonia, the post-IAV lung is immunologically distinct in that IAV infection depletes and impairs resident alveolar macrophages, induces epithelial type I-interferon signaling that skews subsequent cytokine production toward an IFN-γ-dominant profile, and leaves recruited neutrophils with diminished bactericidal capacity yet heightened release of reactive oxygen species(5–8). Clinical studies of acute respiratory distress syndrome (ARDS) have shown that certain antibiotic classes, such as macrolides, tetracyclines, and oxazolidinones, have immunomodulatory activity and can improve ARDS symptoms beyond their antibacterial activity (9). For example, animal studies have shown that linezolid can reduce lung damage and improve survival in IAV-MRSA pneumonia infection (8, 10–12).

In addition to causing excessive inflammation, MRSA produces multiple exotoxins that contribute to the disease. For instance, Panton-Valentine leukocidin (PVL) induces lung epithelial damage in animal models of MRSA pneumonia (13) and in a human cell culture model of IAV-MRSA infection (14). Remarkably, PVL is produced by nearly every strain of MRSA that causes IAV-MRSA pneumonia (15–17). A second *S. aureus* exotoxin, alpha-hemolysin (Hla), has also been associated with virulence in animal models of IAV-MRSA pneumonia (12, 18) and has been linked to severe cases of pneumonia in pediatric patients (19). Although the precise role of these toxins during IAV-MRSA pneumonia infection remains somewhat unclear, it is widely accepted that reducing toxin production using protein synthesis inhibitor antibiotics is beneficial.

Linezolid (a protein synthesis inhibitor) or vancomycin (a cell wall synthesis inhibitor) is recommended by the Infectious Diseases Society of America for the treatment of MRSA pneumonia (20, 21). Clinical and retrospective studies have demonstrated equivalence (22, 23) or superiority of linezolid (24, 25) for the treatment of MRSA-pneumonia, but to our knowledge, there has been no clinical trial evaluating the efficacy of linezolid or vancomycin for the treatment of IAV-MRSA pneumonia. In animal models of IAV-MRSA pneumonia, linezolid and vancomycin each reduce bacterial burden and increase survival, but linezolid attenuates lethal lung damage, reduces inflammation, and lowers MRSA-toxin levels more effectively than vancomycin (10–12). Despite their effectiveness in humans and experimental animals, linezolid and vancomycin are not without limitations. For instance, linezolid has a simplistic dosing strategy that, in certain patient populations, can lead to drug toxicity or to therapeutic failure (26). Linezolid can also cause gastrointestinal symptoms and serious drug interactions with other commonly used pharmaceuticals (26–28). Vancomycin does not reduce toxin production, penetrates lung tissue poorly, and can cause nephrotoxicity (29–31). Thus, additional antibiotic options for the treatment of MRSA and IAV-MRSA pneumonia are greatly needed.

Omadacycline is a semi-synthetic, protein synthesis inhibitor antibiotic belonging to the tetracycline class (32). It was approved by the FDA in 2018 for the treatment of adult community-acquired bacterial pneumonia (CABP) and acute bacterial skin and skin-structure infections (ABSSSI) and is available in both intravenous and oral formulations (33–35). Omadacycline exhibits activity against a broad range of Gram-positive and Gram-negative bacteria, including MRSA (36). Notably, tetracycline-class drugs are known to have anti-inflammatory properties (37), and recent studies have demonstrated that omadacycline dose-dependently suppressed cytokine production from lipopolysaccharide (LPS)-stimulated human monocytes *in vitro*, inhibited neutrophil chemotaxis, and reduced inflammation in a murine model of LPS-induced acute lung injury (38, 39). Thus, we hypothesized that omadacycline could mitigate IAV-MRSA pneumonia in several ways: (i) modulation of cytokine production, (ii) suppression of extracellular bacterial toxins, and (iii) antibacterial activity. Accordingly, the purpose of this study was to evaluate the efficacy and anti-inflammatory properties of omadacycline in the treatment of experimental IAV-MRSA pneumonia.

## RESULTS

### Minimum inhibitory concentration values of antibiotics used in this study

The minimum inhibitory concentration (MIC) values for omadacycline and linezolid against the MRSA USA300 LAC strain used here were determined following Clinical and Laboratory Standards Institute (CLSI) guidelines. Consistent with previous reports as well as CLSI and the Food and Drug Administration (FDA) breakpoints for other *S. aureus* strains (40–42), the LAC strain was susceptible using FDA ABSSSI breakpoints for *S. aureus* (including MRSA) or intermediate using FDA CABP breakpoints for *S. aureus* (methicillin-susceptible isolates only) to omadacycline, and susceptible to linezolid (Table S1).

### Omadacycline treatment increased survival in murine IAV-MRSA pneumonia

An initial study was conducted to determine the therapeutic dose range of omadacycline for the treatment of IAV-MRSA pneumonia in a murine model. Mice were first infected with IAV and then MRSA 7 days later (day 0). Three hours after MRSA infection, IAV-MRSA infected mice were given either sterile saline (vehicle) by intraperitoneal (IP) delivery, omadacycline (5, 10, or 20 mg/kg) by IP delivery, or linezolid (120 mg/kg) by subcutaneous (SQ) delivery, every 12 hours for a duration of 6 days. The mice were monitored until day 10 post-MRSA infection and evaluated for survival, clinical symptoms of infection, and weight loss.

The doses and route of omadacycline were selected based on pharmacokinetic studies conducted in healthy mice (43) and neutropenic mice infected with *S. aureus* or *Streptococcus pneumoniae* (44, 45). The linezolid dose and route chosen were effective in treating MRSA pneumonia in a murine model (46). For both antibiotics, the doses selected are also relevant to expected human drug ranges (47, 48).

**Fig 1:**
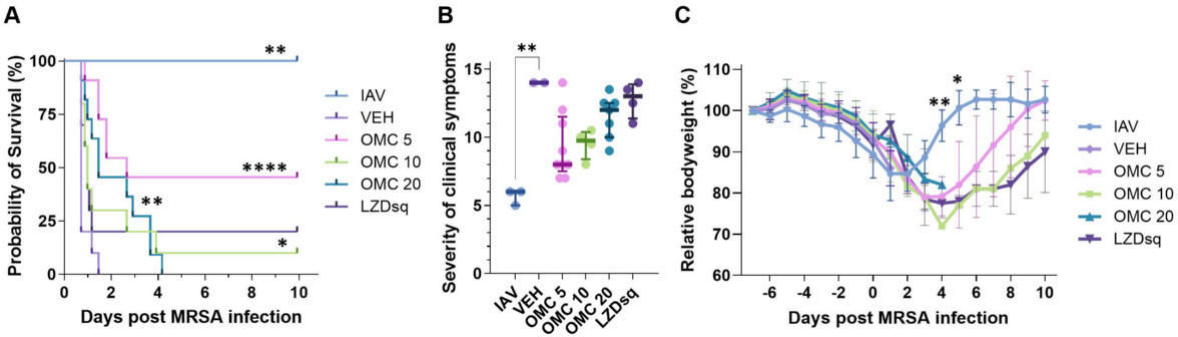
Efficacy of omadacycline and linezolid on IAV-MRSA pneumonia (Experiment 1). IAV-MRSA co-infected mice (*n* = 10 per treatment group, or *n* = 3 for the IAV group) were treated with 5, 10, or 20 mg/kg omadacycline IP (OMC 5, OMC 10, or OMC 20, respectively), 120 mg/kg linezolid SQ (LZDsq), or vehicle IP (VEH). (**A**) Kaplan-Meier estimator of survival after IAV-MRSA infection. (**B**) The sum of the average clinical symptom severity (scruffiness, activity, posture, ocular state; 0 = no symptoms) on day 1 after MRSA infection. Symbols represent individual mice. The line represents the median, and the error bars represent the interquartile range. The level of significance between the VEH-treatment group and all other groups was determined using the Kruskal-Wallis test with Dunn’s multiple comparisons test post hoc. (**C**) Comparison of mean weight changes relative to day −7. Error bars represent standard deviation (SD). Statistical significance was determined by one-way analysis of variance (ANOVA), treatment at each time point compared to OMC 5 by Dunnett’s multiple comparison test post hoc. In all graphs, *P ≤ 0.05, **P ≤ 0.01, ****P ≤ 0.0001; non-significant differences are not shown.

In the vehicle treatment group, all animals succumbed to infection before day 2. All groups treated with antibiotics showed improved survival compared to the vehicle control, but survival reached statistical significance only in mice treated with omadacycline (Fig. 1A). Omadacycline at 5 mg/kg demonstrated the greatest increase in survival at 50% over the 10-day study period. Correspondingly, omadacycline at 5 mg/kg reduced the clinical symptoms more than other treatments, but the difference did not reach statistical significance (Fig. 1B). All IAV-MRSA co-infected mice rapidly lost weight, and survivors began to recover after day 4 (Fig. 1C). During recovery, the 5 mg/kg omadacycline treatment group gained weight more quickly than other groups, though the difference was not statistically significant. All mice in the IAV-only control group survived with minimal symptoms, and weights began to increase on day 1 (8 days after IAV infection).

Plasma concentrations of omadacycline and linezolid were assessed to confirm the drug concentrations used in Experiment 1. Three doses of each drug were administered to healthy BALB/c mice (*n* = 3 per group), with plasma analyzed at 3 hours after the last drug administration. The mean concentrations of omadacycline in plasma were 1.2, 1.4, and 2.6 µg/mL for the 5, 10, and 20 mg/kg doses, respectively (Fig. S1), which agrees with previous findings (43). The mean concentration of linezolid in plasma was lower than expected at 0.019 µg/mL, indicating poor absorption.

**Fig 2:**
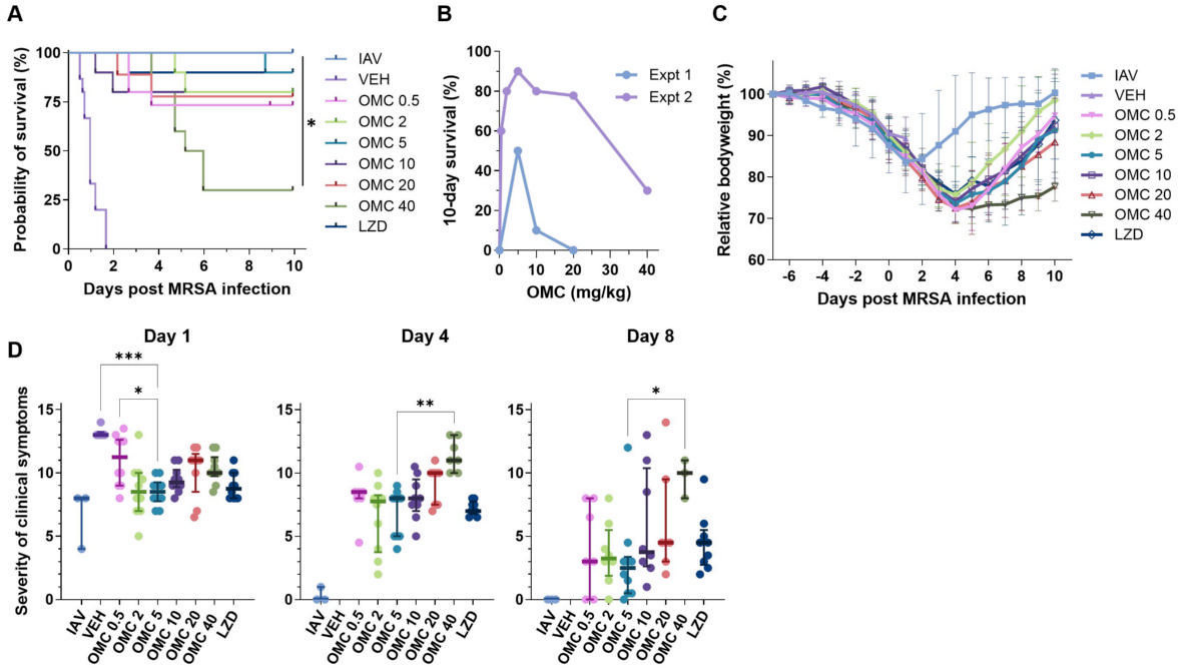
Efficacy of expanded omadacycline dose range (Experiment 2). IAV-MRSA co-infected mice (*n* = 15 for vehicle-treated mice and *n* = 10 per antibiotic treatment group [*n* = 3 for IAV group]) were treated with 0.5, 2, 5, 10, 20, or 40 mg/kg omadacycline (OMC 0.5, OMC 2, OMC 5, OMC 10, OMC 20, or OMC 40, respectively), 120 mg/kg linezolid (LZD), or vehicle (VEH). (**A**) Kaplan-Meier estimator of survival after IAV-MRSA infection. (**B**) Plot of 10-day survival for OMC treatment groups in Experiment 1 (Expt 1) and Experiment 2 (Expt 2). (**C**) Comparison of mean weight changes relative to day −7. Error bars represent SD. Statistical significance was evaluated by one-way ANOVA, each time point was compared to OMC 5 (determined to be the most effective dose in Expt 1) by Dunnett’s multiple comparison test post hoc. (**D**) The sum of the average clinical symptom severity (scruffiness, activity, posture, ocular state; 0 = no symptom) after MRSA infection. Symbols represent individual mice. The line represents the median, and error bars represent the interquartile range. The significance between the VEH-treatment group and all other groups was determined using the Kruskal-Wallis test with Dunn’s multiple comparisons test post hoc. In all graphs, *P ≤ 0.05, **P ≤ 0.01, ***P ≤ 0.001; non-significant differences are not shown.

A second IAV-MRSA pneumonia survival study was conducted using an expanded dose range for omadacycline (0.5, 2, 5, 10, 20, and 40 mg/kg q12h IP). Also, due to the poor absorption of linezolid administered subcutaneously in Experiment 1, linezolid was administered orally (120 mg/kg PO, q12h) in this experiment, herein called Experiment 2, which allowed us to increase the concentration of linezolid administered while maintaining the human-relevant dose. Since different drug delivery methods were used (omadacycline IP and linezolid PO), the vehicle was administered by IP for 10 mice and PO for 5 mice. There were no differences between the two control groups (data not shown), so they were combined for all comparisons to antibiotic treatment groups. As expected, all antibiotic treatments led to a statistically significant increase in survival compared to vehicle control (Fig. 2A). In agreement with Experiment 1, omadacycline at 5 mg/kg improved survival more than the other omadacycline doses tested (Fig. 2B). Orally administered linezolid performed similarly to 5 mg/kg omadacycline (90%, 10-day survival rate), and far better than when administered SQ, as in Experiment 1 (20%, 10-day survival rate). Notably, all antibiotic treatment groups exhibited higher survival rates in Experiment 2 than in Experiment 1, despite only small differences in survival rates in vehicle control groups and initial weights of mice. However, antibiotic administration after MRSA infection was later in Experiment 1 (200 minutes) than Experiment 2 (150 minutes) (Fig. S2), suggesting that a delay in treatment may contribute to worse outcomes.

Also, in line with Experiment 1, animal weights decreased until day 4, with most groups recovering to >90% of their initial body weight by day 10 (Fig. 2C). The only exceptions were the 20 and 40 mg/kg omadacycline treatment groups, which recovered to 88% and 78% of original body weight, respectively. Clinical symptoms were similar across most treatment groups on day 1 post-MRSA infection, with vehicle and 0.5 mg/kg omadacycline treatment groups having the worst symptoms. However, from day 4 to day 8, symptoms in the 40 mg/kg omadacycline treatment group became more severe, while other treatment groups began to recover (Fig. 2D). Notably, injection site sores were observed for animals in the 20 and 40 mg/kg omadacycline treatment groups, suggesting local tissue irritation. Further studies are needed to evaluate the unexpected effects of high-dose omadacycline; however, they are outside the scope of this study.

### Omadacycline reduced toxin production and cytokine production *in vitro*

We next quantified the production of PVL and Hla during exposure to subinhibitory concentrations of omadacycline and linezolid *in vitro* across three time points (9, 12, and 24 hours). Subinhibitory antibiotic concentrations were determined by evaluating bacterial growth in the presence of 1, 1/2, 1/4, 1/8, and 1/16 the MIC of each antibiotic (Fig. S3). Subinhibitory concentrations are defined as any drug concentration that does not inhibit bacterial growth by more than 2-log at any time point (35). PVL and Hla in culture supernatants were quantified by enzyme-linked immunosorbent assay (ELISA) and rabbit erythrocyte lysis assay, respectively.

**Fig 3:**
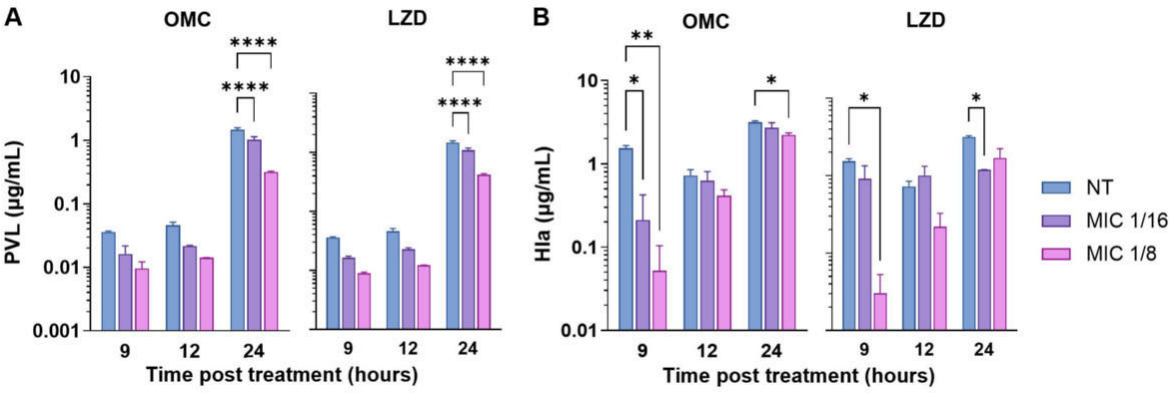
Effects of subinhibitory antibiotics on MRSA toxin production *in vitro*. MRSA was cultured in the presence of 1/8 or 1/16 the MIC of omadacycline (OMC) or linezolid (LZD), and results were compared to a no-treatment (NT) control. The concentration of PVL (**A**) was determined by ELISA, and Hla (**B**) was determined by rabbit erythrocyte lysis assay. Bars represent the mean of *n* = 3 biological replicates with three technical replicates. Error bars represent the standard error of the mean (SEM). Significance was determined by two-way ANOVA, and the effect of the antibiotic was compared to the NT control of the corresponding time point by Dunnett’s multiple comparisons test post hoc, *P ≤ 0.05, **P ≤ 0.01, ****P ≤ 0.0001; non-significant differences are not shown.

PVL concentrations increased over time in the control group but significantly declined in the presence of omadacycline or linezolid, especially after 24 hours of exposure (Fig. 3A). Hla levels were similar across all time points in the no-treatment groups (Fig. 3B). Hla was significantly reduced by 1/8 or 1/16 the MIC of omadacycline at 9 hours and 1/8 the MIC of omadacycline at 24 hours. Linezolid also significantly reduced Hla production at 1/8 the MIC at 9 hours and 1/16 the MIC at 24 hours.

**Fig 4:**
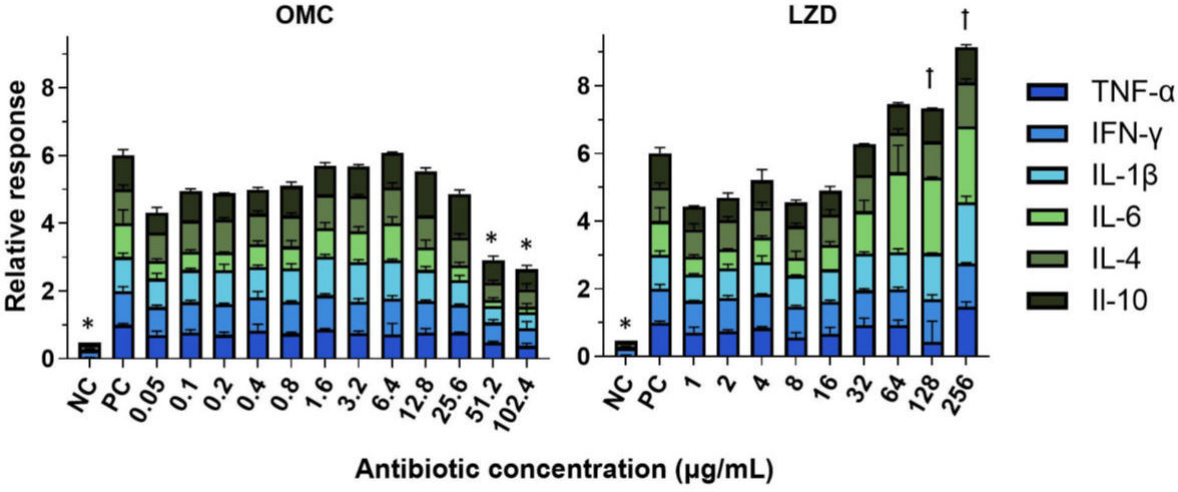
Effects of antibiotics on cytokine production from murine monocytes. Bone marrow monocytes isolated from BALB/c mice (*n* = 2 with two technical replicates) were cultured in the absence of stimulation as a negative control (NC), with LPS stimulation as a positive control (PC), or with LPS plus the indicated concentrations of omadacycline (OMC) or linezolid (LZD). Cytokine levels were scaled relative to the mean of the PC. Bars and error bars are the mean and SD, respectively. Statistics were completed on non-scaled data by one-way ANOVA and compared to PC by Dunnett’s multiple comparisons test post hoc. * or ꝉ indicate the concentrations of antibiotics that statistically (*P* ≤ 0.05) altered the production of all 6 cytokines or 3–5 cytokines, respectively.

Since we previously discovered that omadacycline inhibited the production of cytokines from human monocytes stimulated with LPS (38), we next determined if omadacycline and linezolid had the same effect on mouse monocytes following stimulation with LPS. Based on our work in human cells, six murine cytokines were selected for study: TNF-a, IFN-γ, IL-1b, IL-6, IL-4, and IL-10. All cytokine levels were quantified using a murine multiplex assay. Our results show that higher concentrations of omadacycline (≥51.2 µg/mL) significantly reduced the production of all cytokines tested (Fig. 4 and Table S2). There was also a trend of reduced cytokine production with lower concentrations of omadacycline (0.05 to 0.8 µg/mL), and minimal effects were observed at the mid-range concentrations (0.8 to 25.6 µg/mL). This resulted in a bell-shaped concentration-response curve, most markedly for IL-6 and IL-10, suggesting that there are differential antibiotic effects on cellular signaling pathways at different concentrations. Linezolid also reduced cytokine production at low concentrations (1 to 16 µg/mL), but increased cytokine production, occasionally more than LPS alone, at higher concentrations (32 to 256 µg/mL) (Fig. 4 and Table S2). None of the antibiotic concentrations tested had any effect on monocyte viability (data not shown).

### Omadacycline limited toxin production and modulated the immune response in IAV-MRSA co-infected mice

To understand how omadacycline improves survival from IAV-MRSA-pneumonia *in vivo*, serum, bronchiolar lavage fluid (BALF), lungs, and spleens were collected 8, 24, and 48 hours post-MRSA infection and assessed for microbial burden, MRSA toxins, lung damage, and immunomodulation.

### Effects of omadacycline on microbial burden and bacterial toxin production

**Fig 5:**
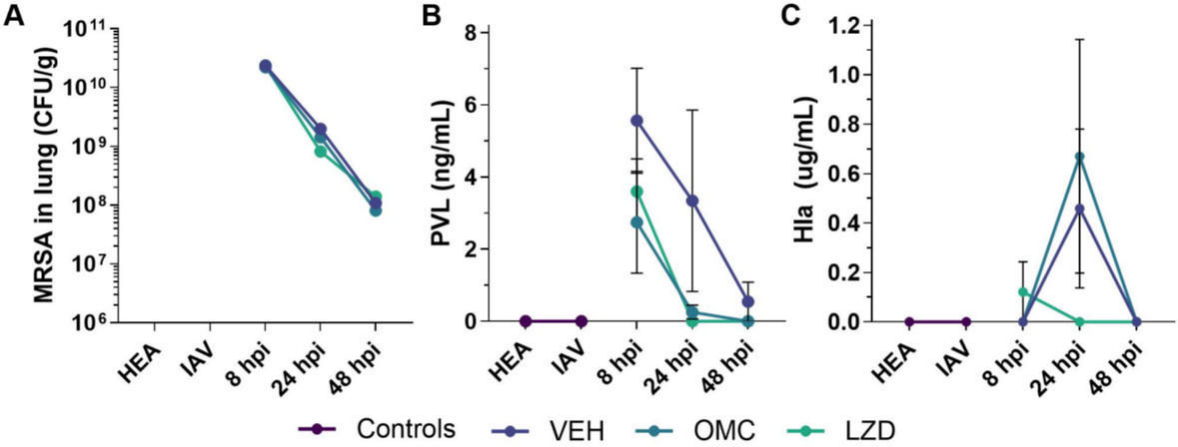
Effects of omadacycline and linezolid on bacterial burden and toxin production in IAV-MRSA-infected mice. The lungs of IAV-MRSA co-infected mice were evaluated for bacterial burden (**A**), PVL (**B**), and Hla (**C**). Data are from *n* = 5 per group per time point. Symbols represent the mean. Error bars represent SEM. Statistical significance was determined by two-way ANOVA and compared to VEH by Dunnett’s multiple comparisons test post hoc. HEA, healthy; IAV, influenza-only infected; VEH, vehicle; OMC, omadacycline; LZD, linezolid.

Omadacycline and linezolid did not impact bacterial populations in lung tissue (Fig. 5A), consistent with previous research on linezolid and other antibiotics in similar IAV-MRSA pneumonia models (12). Similarly, omadacycline and linezolid had no effect on the abundance of live IAV as determined by Tissue Culture Infectious Dose 50 (TCID50; Fig. S4). No bacteria were found in the spleen at any time point (data not shown).

PVL was present in BALF of all groups by 8 hours post-MRSA infection (Fig. 5B). At 8 and 24 hours, PVL levels were substantially lower in omadacycline and linezolid-treated animals than in the untreated control group, but they did not reach statistical significance. Detection of Hla was highly inconsistent (Fig. 5C), likely due to the low sensitivity of the hemolysis assay. Additionally, omadacycline and linezolid did not show a clear impact on LDH, albumin, or total protein in BALF (data not shown).

### Effects of omadacycline on the cellular host response

**Fig 6:**
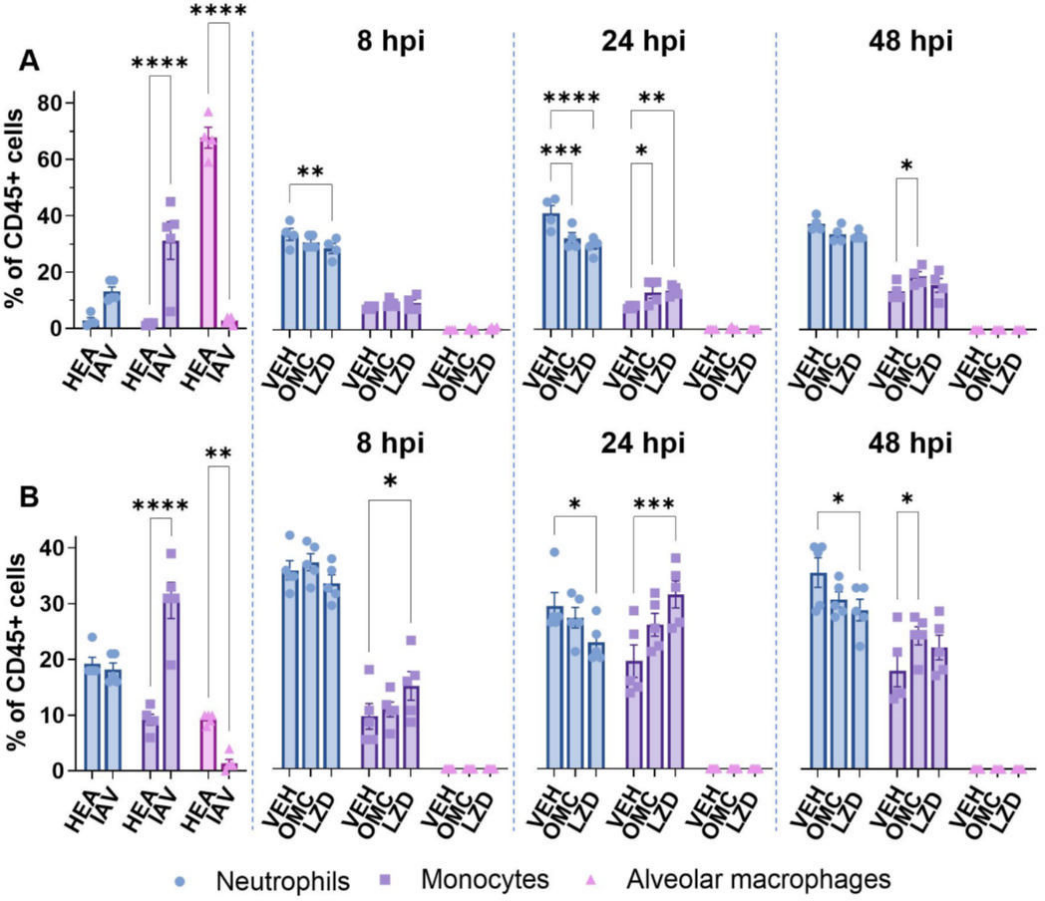
Effects of omadacycline and linezolid on immune cell abundance in lungs after IAV-MRSA infection. Immune cells (CD45+) from (A) BALF and (B) dissociated left lung tissue (*n* = 5 per group per time point) were quantified by flow cytometry. Hours post-infection (hpi) are indicated. Bars indicate the mean for neutrophils (CD45+, CD11b+, CD11c±, Ly6C+, Ly6G+), monocytes (CD45+, CD11b+, CD11c±, Ly6C+, Ly6G−) and alveolar macrophages (CD45+, CD11b±-, CD11c++, Ly6C+, SiglecF+). Symbols represent individual mice, and error bars represent the standard deviation. Statistical significance was determined by two-way ANOVA and compared to VEH by Sidak’s multiple comparisons test post hoc, *P ≤ 0.05, **P ≤ 0.01, ***P ≤ 0.001, and ****P ≤ 0.0001. Non-significant differences are not shown. HEA, healthy; IAV, influenza-only infected; VEH, vehicle; OMC, omadacycline; LZD, linezolid.

IAV infection alone significantly increased monocyte abundance at 7 days in both BALF and lung tissue (Fig. 6, gating strategy in Fig. S5). Additionally, alveolar macrophages were severely diminished by IAV infection, as has been described by others (49). In the IAV-MRSA co-infection model, neutrophil levels increased while monocytes decreased in BALF and lung tissue by 8 hours post-MRSA infection in vehicle-treated animals. At 24 hours post-MRSA infection, omadacycline and linezolid significantly decreased neutrophil infiltration, while increasing monocyte infiltration in both BALF and lung tissue (Fig. 6). By 48 hours post-MRSA infection, differences in the neutrophil population were negligible in BALF but still reduced in lung tissue. Also, at 48 hours, monocytes remained elevated in the omadacycline treatment group in both BALF and lung tissue. Neither time nor antibiotic treatment had any effect on the abundance of alveolar macrophages.

### Effects of omadacycline on circulating and pulmonary cytokine levels

**Fig 7:**
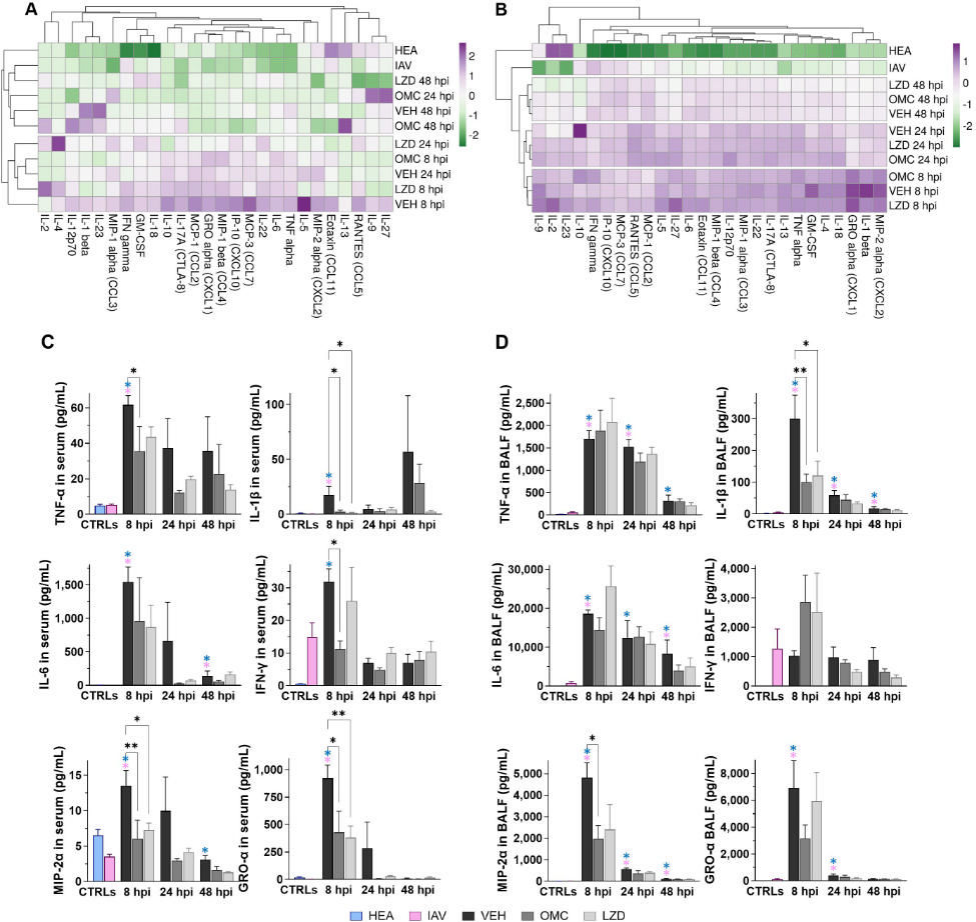
Effects of omadacycline and linezolid on cytokine production. Hierarchical clustering with a heatmap of ln(x)-transformed cytokine values in serum (*n* = 6–7) (A) and BALF (*n* = 4–5) (B). Dendrograms represent clusters using correlation distance and average linkage. Select serum (C) and airway (D) cytokines over time. Bars represent the mean. Error bars represent SEM. In all graphs, statistical significance was determined by one-way ANOVA and compared to VEH by Dunnett’s multiple comparisons test post hoc. Black asterisks are *P ≤ 0.05, **P ≤ 0.01, ***P ≤ 0.001, ****P ≤ 0.0001. Blue and pink asterisks are *P* ≤ 0.05 compared to healthy or IAV controls, respectively. In all figures, hpi is indicated. CTRLs, controls; HEA, healthy; IAV, influenza-only infected; VEH, vehicle; OMC, omadacycline; LZD, linezolid.

Hierarchical cluster analysis was conducted to identify treatment groups with similar cytokine profiles. In serum, there were no tightly clustered cytokines or treatment groups, but two distinct clusters emerged. These included treatment groups with higher overall cytokine levels (8- and 48-hour vehicle, 8- and 24-hour linezolid, and 8-hour omadacycline) and those with lower overall cytokine levels (healthy, IAV-only, 48-hour vehicle, 48-hour linezolid, and 24- and 48-hour omadacycline) (Fig. 7A), suggesting that the second cluster represented recovering animals. In BALF, treatment groups clustered tightly by time post-MRSA infection (8, 24, or 48 hours; Fig. 7B), indicating that cytokine profiles in lung fluid changed with time more so than treatment.

To assess specific cytokine changes, the concentration of each cytokine was compared between treatment groups at each time point. At the 8-hour time point, most pro-inflammatory cytokines in serum and BALF were significantly increased after MRSA infection compared to IAV-only and healthy controls (Fig. S6). Consistent with previous findings in the IAV-MRSA model (10, 11), TNF-α, IL-1β, IL-6, MIP-2, and GRO-α (mouse KC) were significantly increased in serum and BALF after MRSA infection, while IFN-γ levels were relatively unaffected (Fig. 7C and D). Omadacycline treatment reduced most serum cytokines 8 hours post-MRSA infection, including TNF-α, IL-1β, MIP-2, GRO-α, and IFN-γ. In contrast, linezolid only reduced eight cytokines in serum, all at the 8-hour time point, and increased GM-CSF, IL-2, IL-4, and IL-18 levels in serum at the 24- to 48-hour time points (Fig. S6). In BALF, omadacycline reduced three cytokines, IL-1β, GM-CSF, and MIP2, at the 8-hour time point (Fig. 7D). At this same time point, linezolid reduced only IL-1β and increased IL-27. Neither omadacycline nor linezolid significantly affected BALF cytokine levels at the 24- or 48-hour time points.

### Histopathology

Images of hematoxylin and eosin (H&E)-stained lungs revealed localized patches of inflammation 7 days after infection with IAV, which became more abundant and widespread following MRSA infection (Fig. S7A). Over time, inflammation was less patchy and more dispersed, particularly in groups receiving antibiotic treatment. Microscopically, IAV-infected lungs were filled mainly with lymphocytes, whereas the alveolar spaces of IAV-MRSA-infected lungs were filled mostly with neutrophilic exudate (Fig. S7B). There were no clear pathological differences between the treatment groups. Additionally, pathological scoring of chronic, subacute, and acute inflammation, edema of the pleural, and fibrin formation indicated no significant differences between treatment groups (data not shown).

## DISCUSSION

MRSA pneumonia following IAV infection carries substantial morbidity and mortality, and new antibiotic treatments are needed. Omadacycline has demonstrated non-inferiority to linezolid for the treatment of human skin and soft tissue infections caused by MRSA (34) and non-inferiority to moxifloxacin for the treatment of community-acquired bacterial pneumonia (33, 35), but no studies on the effect of omadacycline on IAV-MRSA pneumonia have been conducted. In this current study, we examined the efficacy of omadacycline for the treatment of IAV-MRSA pneumonia co-infection in a mouse model. In two separate survival studies, we found that 5 mg/kg of omadacycline administered every 12 hours was the most effective dose. This dose resulted in exposures in mice that closely matched the human exposures achieved with the US FDA-approved dose regimens, as well as those of previously published pharmacokinetic studies in mice (43, 45, 50, 51). Additionally, omadacycline at 5 mg/kg was equally effective as 120 mg/kg linezolid delivered orally (Experiment 2) but far outperformed linezolid when linezolid was delivered subcutaneously (Experiment 1). Although a more complete pharmacokinetic analysis is needed, we believe that the poor performance of linezolid delivered subcutaneously is due to slow absorption of the large volume needed (0.8–1 mL) to administer 120 mg/kg of linezolid (solubility of linezolid is ~2 mg/mL), which was evident in the low serum concentration 3 hours post-administration in healthy mice. However, we cannot rule out that the difference in linezolid efficacy was a result of slight variances between Experiment 1 and Experiment 2, especially the time of antibiotic administration after MRSA infection, which differed by 50 minutes. Indeed, a previous study in a similar model found that antibiotic treatment delivered 4 hours post-MRSA infection was too late to rescue animals from IAV-MRSA pneumonia-mediated death (12).

For insight into the improved survival attributed to omadacycline and linezolid, we investigated their effects on MRSA-toxin production, antimicrobial activity, and immune modulation, both *in vitro* and *in vivo*. As protein synthesis inhibitors, omadacycline and linezolid produced the anticipated outcome of reducing PVL and Hla toxin production *in vitro*, as well as a reduction of PVL *in vivo*. While few studies have investigated the impacts of antibiotics on MRSA-toxin production during IAV-MRSA infection, one study found that infection of a *Δhla* MRSA mutant reduced production of IL-6 and acute lung damage in IAV-MRSA co-infected animals and that linezolid was more effective than vancomycin in preventing these Hla-like pathological changes (12). This suggests that linezolid improved survival by reducing Hla production; however, Hla production was not measured, so it remains unclear if linezolid directly reduced Hla production.

Clinical studies report that nearly all strains of *S. aureus* that cause post-influenza (or post-influenza-like) MRSA pneumonia produce PVL (15–17). Since neutrophils are the primary target of PVL (52) and PVL-induced neutrophil lysis leads to the release of proteases and/or reactive oxygen species (8, 14), PVL production can indirectly result in severe lung damage. Here, omadacycline and linezolid reduced airway PVL production, but they did not reduce airway damage. These findings suggest that PVL-induced neutrophil lysis did not play a significant role in this model. This conclusion is supported by the fact that the action of PVL is limited to certain species, such as humans and rabbits, but not mice (52). Therefore, the observed improvement in survival may be partially due to reduced PVL production, which would be particularly important in humans, but the precise role of PVL in the mouse model of IAV-MRSA pneumonia remains uncertain.

Many studies have demonstrated the positive impact of immunosuppressive antibiotics in treating ARDS (reviewed in reference 9), and it has been shown that excessive immune activity contributes to lung damage and mortality during IAV-MRSA pneumonia in the murine model (53, 54). Some studies even suggest that immunomodulation is more crucial than reducing bacterial burden, as immunomodulatory antibiotics can have little effect on bacterial burden and still improve outcomes (12). Similarly, in this study, omadacycline or linezolid treatment improved survival while having no effect on bacterial burden in the lungs. Still, this finding is surprising, since omadacycline and linezolid have been shown to have good distribution to the lungs (45, 46), and it contrasts with some IAV-MRSA and MRSA-only pneumonia models, in which omadacycline and/or linezolid have been shown to significantly reduce bacterial burden (11, 21, 44, 55, 56). This contrast could be due to a variety of factors, such as differences in inoculum size, IAV and MRSA strains, timing of the MRSA infection, mouse strain, or route of antibiotic exposure. The lack of bacterial burden reduction despite improved survival may reflect IAV-induced structural lung damage, impaired host immune cell function, and increased alveolar edema, potentially contributing to reduced antibiotic penetration and bacterial clearance. Additionally, protein synthesis inhibitors exert therapeutic effects by limiting bacterial toxin production and modulating host inflammatory responses; therefore, immunomodulation and toxin production may have greater clinical relevance than bacterial burden reduction in the IAV-MRSA pneumonia model.

The anti-inflammatory effects of omadacycline are supported by our data on immune cell and cytokine dynamics. *In vitro*, omadacycline treatment decreased cytokine production in both human (THP-1 and freshly isolated serum monocytes) (38, 39) and murine LPS-stimulated monocytes, suggesting the immune response observed in the murine model is similar to the human immune response to omadacycline. Interestingly, linezolid increased cytokine production at high concentrations *in vitro* and at later time points *in vivo* compared to the untreated control group, suggesting there are differences in the immunomodulatory properties of omadacycline and linezolid, although the consequences of these differences remain to be elucidated. Both antibiotics decreased the abundance of neutrophils in the BALF and lung tissue, especially at the 24-hour time point, suggesting an attenuation of the exaggerated immune response typically seen in IAV-MRSA infections. This finding is consistent with the immunomodulatory effects of linezolid, reported by others (10–12), and correlates with our finding that both antibiotics reduced multiple airway cytokines known to play a role in neutrophil recruitment in acute lung injury (57, 58). In corroboration with this data, production of TNF-α, IL-1β, and IL-6 was reduced in the lungs of mice treated with omadacycline in an LPS-induced murine lung injury model (ALI) (39).

Previous studies have reported that the persistent infiltration of neutrophils can exacerbate lung damage and lead to poor outcomes in IAV-MRSA pneumonia (8, 11). Interestingly, reduced neutrophil recruitment was not associated with reduced lung damage in this study. Similarly, in the ALI model, treatment with omadacycline was associated with reduced white blood cells and neutrophil recruitment in the lungs, although there was no change in overall lung injury severity (39). The observed lack of association between reduced neutrophil recruitment and diminished lung injury in our study and others may reflect that once substantial epithelial injury is established, subsequent reduction in neutrophil recruitment alone may be insufficient to reverse or substantially diminish tissue damage. Thus, further work is needed to fully understand how changes in airway inflammation caused by omadacycline and linezolid contribute to improved outcomes in this model.

In conclusion, our study demonstrates that omadacycline was efficacious in treating post-influenza MRSA pneumonia in a murine model. Omadacycline, at a potentially clinically relevant dose of 5 mg/kg given twice daily in mice, proved equivalent to 120 mg/kg oral linezolid. Interestingly, in this infection model, the benefit of omadacycline and linezolid appeared to be largely independent of bacterial burden reduction and primarily dependent upon their immunomodulatory effects. This study provides the first preclinical evidence of omadacycline efficacy against IAV-MRSA pneumonia. Future research is needed to dissect the complex interplay of cytokine modulation and immune cell dynamics that mediate the therapeutic efficacy observed with omadacycline and linezolid in this murine model of severe respiratory infection.

## MATERIALS AND METHODS

### Minimum inhibitory concentrations

The USA300 CA-MRSA LAC strain, used for all experiments in this study (a gift from Dr. Frank R. DeLeo, NIH), was compared to the MRSA Wichita strain (ATCC 29213) as a susceptibility control, as suggested by the Clinical and Laboratory Standards Institute (CLSI guidelines). Pharmaceutical-grade omadacycline was provided by Paratek Pharmaceuticals (King of Prussia, PA). Pharmaceutical-grade linezolid was purchased from the VA Medical Center Pharmacy (Boise, ID). The MIC of each antibiotic for each strain of *S. aureus* was determined by the microbroth dilution, according to CLSI guidelines. Briefly, isolated colonies were used to inoculate overnight cultures. The next day, *S. aureus* was sub-cultured and grown at 37°C in 5% CO_2_ with shaking (200 rpm) to mid-log phase. Bacteria were harvested by centrifugation, washed in sterile saline, and seeded in 96-well flat-bottom plates at 5 × 10^5^ CFU per milliliter in the presence of antibiotic in freshly made cation-adjusted Muller Hinton Broth II (CAMHB II). Antibiotics were solubilized and diluted in water. Plates were incubated at 37°C in 5% CO_2_ for 24 hours, at which point growth inhibition was determined by optical density (O.D. 600). The MIC is defined as the lowest antibiotic concentration that inhibited measurable bacterial growth (Table S1).

### IAV-MRSA co-infection model

All animal experiments were approved by the Boise VA Medical Center’s Institution Animal Care and Use Committee and conducted in the Boise VA Medical Center’s AAALAC-accredited Veterinary Medical Unit. Specific-pathogen-free BALB/c mice were purchased from Charles River Laboratories (Hamilton, CA). For survival studies, wild-type 6-week-old female BALB/c mice (*n* = 10–15 per group) were anesthetized with ketamine and xylazine (KX) cocktail (90 mg/kg Ketamine and 12 m/kg Xylazine) delivered IP and 150 PFU of mouse-adapted Influenza A/Puerto Rico/8/34 (PR8; H1N1) (ATCC VR-1469) was delivered in 50 µL sterile saline via intranasal inoculation. Seven days later, 6.9 × 10^8^ CFU (Experiment 1) or 6.7 × 10^8^ CFU (Experiment 2) washed late-log phase CA-MRSA LAC strain was administered intranasally in 50 µL using the same technique. Immediately after infection, anesthetized mice were placed on ~37°C heating pads until they were active (~30–45 minutes) before being placed back into cages. Mice were given omadacycline (0.5–40 mg/kg IP), linezolid (120 mg/kg SQ or PO), or vehicle q12h (IP or PO), 2 or 3 hours after MRSA infection. Antibiotic treatment continued for 6 days or until mice were euthanized. IAV-MRSA-infected mice were also given 0.1 mg/kg buprenorphine every 12–72 hours as needed (Rickitt Benckiser Pharmaceuticals Inc. or ZooPharm). Mice were weighed daily and scored for signs and symptoms of infection and distress three times per day. Four criteria (activity, fur aspect, eye opening, and posture) were assessed and scored 0 to 3 or 4 (no symptom to worst symptom) in severity, as discussed in previous studies (59, 60). A score of ≥14 dictated the decision for euthanasia.

The doses and route of administration of omadacycline were selected based on pharmacokinetic studies conducted in healthy mice (43) and neutropenic mice infected with *S. aureus* or *Streptococcus pneumoniae* (44, 45). The linezolid dose, delivered SQ, was chosen because it was shown to be effective in treating MRSA pneumonia in a murine model (46). The decision to switch the administration route but maintain the dose was supported by a previous study showing the PK for linezolid SQ versus PO delivery is similar (61). For both antibiotics, the doses selected were also relevant to expected human drug ranges (47, 48).

For the collection of tissues and serum over time, 7 mice per group per time point were infected as above but with 4.0 × 10^8^ CFU MRSA. Mice were given 5 mg/kg omadacycline IP, 120 mg/kg linezolid PO, or vehicle IP q12h, 2 hours after MRSA infection. From five mice per group, serum, BALF, BALF cells, lungs, and spleens were collected at 8, 24, and 48 hours post-MRSA infection, as detailed below. Controls included healthy, non-infected mice and mice infected with IAV-only. Samples from IAV-only infected mice were collected 7 days after IAV infection. Serum was evaluated for cytokines. The right lungs and spleens were evaluated for microbial (IAV and MRSA) or bacterial burden, respectively. BALF was evaluated for bacterial toxins, lung injury (LDH, albumin, and total protein), cellular infiltrate, and cytokines. The left lungs were used for the quantification of cellular infiltrate. From two mice per group, whole lungs were taken for pathological assessment.

### Determination of antibiotic levels in plasma

Healthy 6-week-old female BALB/c mice (*n* = 3/group) were given three doses of omadacycline at 5, 10, or 20 mg/kg IP, linezolid 120 mg/kg SQ, or 0.1 mL sterile saline IP, q12h. Three hours after the last antibiotic dose, mice were put under anesthesia with a KX cocktail. Blood was collected by cardiac puncture using a 1 mL syringe filled with 0.05 mL sterile 0.5 M EDTA. Whole blood was put into an EDTA blood collection tube and inverted 10 times. After blood collection, the EDTA tubes were centrifuged at 3,000 rpm for 10 minutes at 4°C. The plasma was collected and placed into 1.5 mL microcentrifuge tubes and immediately frozen at −70°C.

Omadacycline, linezolid, and urea were extracted from plasma via acetonitrile (ACN) protein precipitation. 100 µL of plasma was mixed with 500 µL of ACN and 100 µL of internal standard (IS) and centrifuged for 15 minutes. The supernatant layer was collected in a glass tube and dried under nitrogen gas. The dried sample was reconstituted in water, transferred to a sample vial, and 20 µL injected into the liquid chromatography with tandem mass spectrometry system. A Luna C18 column achieved the chromatographic separation of omadacycline, linezolid, and urea (75 mm × 2 mm, 3 µm particle size) protected with a Phenomenex (CA, USA) guard column, which was operated at 40°C. The mobile phase was composed of 10 mM ammonium formate in water (A) and ACN (B). Omadacycline and linezolid were detected using positive electrospray ionization (ESI) sources. Mass analysis was carried out in multiple reaction monitoring modes with the transition of parent ions to the product ions. The calibration curve was prepared by spiking 250 ng/mL of omadacycline-D9 with a final concentration of 50–1,600 ng/mL of omadacycline and 125–2,000 pg/mL of linezolid, respectively. The analysis of urea was conducted using Q1 mode, and the [M+1]^+^ ion of urea at m/z of 61.039 was detected in the ^+^ESI mode. The calibration curve for urea was 18–500 ng/mL. The methods were validated for their accuracy and precision. For each analyte, the area under the mass transition peak was calculated and divided by the area under the mass transition peak for the internal standard. The resulting values were used to construct the standard curves. The concentration of each analyte in plasma samples was calculated by extrapolation using the relevant standard curve.

### MRSA toxin production

MRSA LAC strain cultures were started by diluting overnight cultures ~1/800 to provide a starting concentration of 5 × 10^5^ CFU/mL in 15 mL of CAMHB II. At time 0, antibiotics were added to give a final concentration of 1, 1/2, 1/4, 1/8, or 1/16 of the MIC. Additionally, PBS served as a no-treatment (NT) control. All cultures were incubated while shaking (200 rpm) at 37°C. At 0, 9, 12, and 24 hours post-inoculation, 1 mL samples were removed from each culture for quantitative dilution plating and analysis of toxin production (PVL-LukS and Hla) from culture supernatants.

PVL was quantified by ELISA, as we have previously described (29). Briefly, enzyme immunoassay/radioimmunoassay plates were coated with 1 µg/mL anti-PVL mAb 1D9 (IBT Bioservices, Gaithersburg, MD) in 50 mM carbonate buffer (pH 9.6). Capture antibody was removed, and plates were blocked with PBS + 5% skim milk overnight at 4°C. Tag-free LukS-PV standard (0.8–50 ng/mL; IBT Bioservices) and diluted culture supernatants (1:200 to 1:800) were applied to ELISA plates for 2 hours at 37°C. Plates were washed with PBS-Tween (0.05%) and successively incubated with 0.25 µg/mL rabbit polyclonal anti-PVL (LukS) (IBT Bioservices) and HRP-linked goat anti-rabbit IgG (Thermo Scientific). After washing, assays were developed with 1-step Ultra TMB (Thermo Scientific).

Hla activity was assayed by a standard rabbit erythrocyte lysis assay, as described previously (29). In brief, sterile-filtered culture supernatants were diluted in PBS (1:10 to 1:80) in a microtiter plate, and an equal volume of washed rabbit erythrocytes (2% in PBS) was added. Sterile deionized water was included as a 100% hemolysis control. After incubation for 1 hour at 37°C, plates were centrifuged, supernatants were transferred to a new microtiter plate, and the absorbance was read at 550 nm.

### Antibiotic-induced cytokine production and cytotoxicity

Primary bone marrow monocytes were collected aseptically from two healthy BALB/c mice after euthanasia, as previously described (62, 63), with modifications. Briefly, whole femurs and tibias of two mice were removed, and bone marrow was collected by centrifugation (10,000 × *g* for 30 seconds at 4°C). Bone marrow was resuspended in 1 mL ammonium-chloride-potassium lysis buffer (Miltenyi), incubated at room temperature for 2 minutes, then filtered through a 0.70 µm filter and washed with 10 mL of PBS into a 50 mL conical tube. The cell suspension was washed 2× in PBS (350 × *g* for 4 minutes at 4°C) and counted. Twelve million cells were suspended in 20 mL of supplemented macrophage media (DMEM high glucose, no sodium pyruvate, GlutaMax, Pen/Strep, 10% FBS, and 50 ng/mL recombinant mouse M-CSF [Fisher Scientific]), plated in 15 cm non-tissue culture treated plates, and incubated at 37°C 5% CO_2_. Three days later, 20 mL of supplemented macrophage media was added, and adherence was confirmed by microscopy on day 5. On day 5, the media was removed, and cells were washed with 5 mL sterile PBS. Cells were carefully scraped off the dish using a cell scraper in 5 mL of macrophage media. Cells were resuspended in supplemented macrophage media at 0.5 million cells/mL and plated onto tissue culture-treated 96-well plates at 50,000 cells per well and incubated overnight.

Antibiotics were prepared in water at 50 mg/mL of omadacycline or 1 mg/mL linezolid, filtered to 0.2 µm, then diluted into macrophage media to 50% the final concentration. Water without antibiotics was prepped identically in parallel as vehicle controls. Media was removed from 96-well plates containing adherent monocytes, and 100 µL of fresh macrophage media and 100 µL of antibiotic (or vehicle) was added. Experiments were done in duplicate. Two hours later, 2 µL (200 pg) of LPS (Fisher Scientific Cat. No. 50-112-2025) was added and incubated for 24 hours at 37°C. Supernatants were then collected for cytokine analysis, and cells remaining on the plate were analyzed for viability.

A 100 µL mixture of 5 µM calcein AM and 10 µM ethidium homodimer-1 (LIVE/DEAD Viability/Cytotoxicity Kit, Thermo #L3224) in PBS was added to the cells in the 96-well plate. The plate was incubated at room temperature for 45 minutes. Fluorescence was read at 530 and 645 nm.

Cytokines from culture supernatants were quantified using a multiplex assay (Luminex). Briefly, two 25 µL samples were aliquoted into a 96-well preconfigured ProcartaPlex Mouse Cytokine and Chemokine Panel, 6-plex (Thermofisher). Cytokines were analyzed on the MAGPIX Luminex 200 multiplex plate reader and quantified using xPOTENT Software Solutions for Luminex Systems.

### Collection of biological samples

For the collection of serum, mice were put under anesthesia with KX cocktail, and blood was collected by cardiac puncture. Blood was clotted at room temperature for 1 hour before centrifugation (10 minutes at 1,000 × *g*). The recovered serum was collected in a 1.5 mL microcentrifuge tube and put on ice for ~2 hours, then frozen at −70°C until analysis was conducted.

BALF was collected by inserting a 22-gauge catheter needle into the trachea of exsanguinated mice. Once the catheter needle was in place, the needle was removed, and a 1 mL syringe with 0.5 mL sterile PBS was attached to the end of the catheter. PBS was slowly injected and aspirated three times. Lungs were observed for proper inflation and deflation during injection and aspiration. The recovered fluid was collected in a 1.5 mL microcentrifuge tube. The PBS injection, aspiration, and collection of BALF were completed a second time. BALF was kept on ice until all samples were collected, then centrifuged for 3 minutes at 200 × *g* at 4°C. The supernatant was transferred to a new microcentrifuge tube and immediately frozen at −70°C. The cell pellet was resuspended in a cell storage buffer (Miltenyi) and stored at 4°C for no longer than 3 days before analysis, as suggested by the manufacturer.

For quantification of bacteria, virus, and tissue-associated immune cells, after BALF collection, the catheter was removed, left (immune cell analysis) and right lungs (bacterial and viral titer) were separated by severing at the left and right bronchus. Spleens were then excised. Lungs and spleens were submerged in a Tissue Storage Buffer (Miltenyi) and stored at 4°C for no longer than 3 days before analysis, as suggested by the manufacturer.

For lung histology, a 22-gauge catheter needle was inserted into the trachea of exsanguinated mice. Once the catheter needle was in place, the needle was removed, and 0.5 mL of formalin was slowly injected into the lungs while observed for proper inflation. Lungs were then tied off at the trachea, just below the catheter insertion site, and submerged in formalin for storage at room temperature for 3 weeks before grossing and embedding.

### Quantification of bacteria and viruses

Lungs and spleens were homogenized using the gentleMACs dissociator with heat (Miltenyi). Tissues were transferred to a MACS M tube with 2 mL of cold PBS and dissociated using the RNA protocol 1. For quantification of bacteria, 100 µL of homogenate was serially diluted in cold PBS, plated in duplicate onto blood agar plates, incubated at 37°C, 5% CO_2_, overnight, and CFUs per milliliter were calculated. For quantification of virus, 1 mL of homogenate was centrifuged at 600 × *g* for 10 minutes, and the supernatant was stored at −80°C until assayed. The TCID50 of IAV was determined by serial dilutions of the virus on Madin-Darby canine kidney cell monolayers, as previously described (64).

### Evaluation of lung damage

BALF samples were assayed for total protein, LDH, and albumin according to the manufacturer’s instructions following the Microplate Procedure from the Micro BCA Protein Assay Kit (Thermo Cat. No. #23235), the CytoTox 96 Non-Radioactive Cytotoxicity Assay (Promega Cat. No. #G1780), and Bethyl Laboratories, Inc. Mouse Albumin ELISA kit (Fisher Scientific Cat. No. NC0096469).

### Quantification of immune cells

For quantification by flow cytometry, BALF cells and dissociated lung tissue cells were incubated in Fc block (TruStain FcX PLUS, BioLegend) for 10 minutes on ice. Fc block was removed, and cells were resuspended in 1:2,000 Zombie Dye plus markers for CD45-AF700, CD11b-PerCP/Cy5.5, CD11c-BV750, Ly-6C-AF488, Ly-6G-PE/Cy7, Siglec-F-APC, MHC II-BV605, CD24-PE, and CD64-BV421 and isotype controls (BioLegend). The cells were incubated on ice, in the dark, for 30 minutes, then washed twice with cold PBS. Cells were resuspended in PBS, transferred to a 96-well plate, and analyzed on a Beckman Coulter Cytoflex and quantified using FlowJo software.

For manual quantification, BALF cells were resuspended in Cytospin Collection Fluid (Thermo Scientific). Cells (2–3 drops) were added to a labeled Shandon Cytofunnel (Thermo Scientific) and spun at 600 × *g* for 10 minutes. Slides were air-dried and then stained using HemaDiff Rapid Stains Set following the manufacturer’s directions (StatLab, McKinney, TX). Differentials were quantified manually by a medical science laboratory technician and a board-certified pathologist.

### Analysis of cytokine production *in vivo*

Frozen serum and BALF collected from healthy, IAV-infected, and IAV-MRSA-infected mice were thawed, and two 25 µL samples were aliquoted into a 96-well preconfigured ProcartaPlex Mouse Cytokine and Chemokine Panel, 26-plex (Thermofisher). Cytokines were analyzed on the MAGPIX Luminex 200 multiplex plate reader and quantified using xPOTENT Software Solutions for Luminex Systems.

The heatmap with hierarchical cluster analysis was performed to examine 26 cytokines over eleven treatment groups using ClustVis (65). Original cytokine values were ln(x)-transformed. Unit variance scaling was applied to rows. Cytokines and treatment groups were clustered based on Euclidean distance and the average linkage.

### Lung histology

Fixed whole lungs were paraffin-embedded, sectioned (4 µm), and stained with hematoxylin and eosin. Sections were imaged by slide scan and observed under the light microscope by a board-certified pathologist to score and assess pathological changes in the lungs.

### Statistics

Statistical significance of Kaplan-Meier survival curves was determined by pairwise comparison to vehicle by log-rank (Mantel–Cox) test. Comparisons between the two study groups were statistically evaluated using an unpaired Mann–Whitney U test. Comparisons between more than two groups (single factor) were evaluated using one-way analysis of variance (ANOVA) with Dunnett’s or the Kruskal–Wallis test with Dunn’s test as indicated in figure legends. Two-way ANOVA was used to evaluate more than two groups at different time points. Unless otherwise noted, statistical significance was quantified in all tests as *P < 0.05, **P < 0.01, ***P < 0.001, and ****P < 0.0001. All statistical analysis was performed using GraphPad Prism 10 software.

## SUPPLEMENTAL MATERIAL

Supplemental material aac.00469-25-s0001.pdf Tables S1 to S3; Fig. S1 to S7.

ASM does not own the copyrights to Supplemental Material that may be linked to, or accessed through, an article. The authors have granted ASM a non-exclusive, world-wide license to publish the Supplemental Material files. Please contact the corresponding author directly for reuse.
