# Supplementary material for: Omadacycline exhibits anti-inflammatory properties and improves survival in a murine model of post-influenza MRSA pneumonia
Source: Antimicrob Agents Chemother. 2025 Aug 4;69(9):e00469-25. doi: 10.1128/aac.00469-25 (PMC12406655; doi:10.1128/aac.00469-25)
Supplement: Supplemental material — Tables S1 to S3; Fig. S1 to S7. [file aac.00469-25-s0001.pdf]

Supplementary Table 1. MICs (µg/mL) of OMC and LZD against MRSA LAC strain

Antibiotic (abbreviation)	MIC	Susceptibility
Omadacycline (OMC)	0.5	S[a]/I[b]
Linezolid (LZD)	4	S[c]

S = susceptible; R = resistant; I = Intermediate

[a] FDA ABSSSI breakpoints

[b] FDA CABP breakpoints

[c] breakpoints according to the Clinical Laboratory Standards Institute

Supplementary table 2. Cytokine production from LPS stimulated murine monocytes after omadacycline or linezolid exposure

omadacycline

	TNF-α		IFN-γ		IL-1β		IL-6		IL-4		Il-10	
µg/mL	mean	SD	mean	SD	mean	SD	mean	SD	mean	SD	mean	SD
NC	0.04	0.01	0.21	0.00	0.04	0.01	0.00	0.00	0.15	0.00	0.03	0.00
PC	1.00	0.04	0.91	0.05	1.03	0.03	0.67	0.01	0.93	0.11	1.11	0.01
0.05	0.70	0.11	0.84	0.05	0.84	0.15	0.51	0.07	0.85	0.00	0.58	0.16
0.1	0.77	0.08	0.91	0.05	0.95	0.04	0.53	0.06	0.93	0.00	0.86	0.07
0.2	0.71	0.07	0.91	0.02	1.00	0.18	0.54	0.01	0.97	0.05	0.78	0.02
0.4	0.83	0.19	0.99	0.18	0.90	0.06	0.67	0.10	0.91	0.08	0.71	0.06
0.8	0.75	0.04	0.94	0.03	0.99	0.13	0.65	0.17	0.91	0.08	0.88	0.12
1.6	0.87	0.02	1.02	0.04	1.13	0.07	0.84	0.17	1.00	0.00	0.85	0.08
3.2	0.76	0.06	0.93	0.08	1.17	0.07	0.91	0.13	1.04	0.05	0.89	0.05
6.4	0.72	0.33	1.06	0.08	1.13	0.03	1.09	0.18	1.06	0.13	1.03	0.01
12.8	0.77	0.12	0.94	0.03	0.93	0.09	0.67	0.21	0.93	0.00	1.32	0.09
25.6	0.77	0.01	0.83	0.01	0.73	0.13	0.44	0.08	0.82	0.11	1.29	0.12
51.2	0.48	0.03	0.60	0.08	0.48	0.00	0.19	0.06	0.52	0.05	0.65	0.12
102.4	0.38	0.07	0.52	0.20	0.48	0.09	0.17	0.09	0.52	0.16	0.59	0.11

linezolid

	TNF-α		IFN-γ		IL-1β		IL-6		IL-4		Il-10	
µg/mL	mean	SD	mean	SD	mean	SD	mean	SD	mean	SD	mean	SD
NC	0.04	0.01	0.21	0.00	0.04	0.01	0.00	0.00	0.15	0.00	0.03	0.00
PC	1.00	0.04	0.91	0.05	1.03	0.03	0.67	0.01	0.93	0.11	1.11	0.01
1	0.71	0.16	0.94	0.01	0.78	0.03	0.52	0.06	0.82	0.16	0.68	0.01
2	0.74	0.04	0.99	0.07	0.88	0.11	0.56	0.01	0.86	0.11	0.66	0.14
4	0.84	0.04	1.00	0.01	0.95	0.18	0.72	0.06	0.89	0.16	0.81	0.31
8	0.56	0.14	0.92	0.04	0.92	0.02	0.53	0.19	0.95	0.08	0.69	0.07
16	0.66	0.20	0.96	0.05	0.96	0.00	0.72	0.10	0.89	0.16	0.71	0.13
32	0.93	0.21	1.04	0.04	1.08	0.06	1.26	0.32	1.07	0.00	0.91	0.01
64	0.92	0.16	1.07	0.06	1.10	0.10	2.36	0.80	1.16	0.13	0.86	0.05
128	0.43	0.61	1.26	0.13	1.37	0.01	2.24	0.02	1.07	0.00	0.98	0.01
256	1.47	0.15	1.29	0.01	1.81	0.18	2.25	0.00	1.29	0.10	1.04	0.07

NC - negative control, monocytes not stimulated with LPS
PC - positive control, monocytes stimulated with LPS

Supplementary Table 3. Median survival after IAV-MRSA infection.

Experiment 1		
Antibiotic	Dose (mg/kg)	Median survival (day)
Influenza-only infection		Undefined
Vehicle		0.71
Omadacycline	5	2.67
Omadacycline	10	0.98
Omadacycline	20	1.46
LinezolidSQ	120	0.98

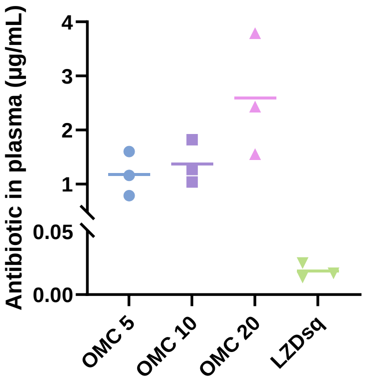

Supplementary Figure 1. Quantification of omadacycline and linezolid in plasma. Mice were given 5 mg/kg, 10 mg/kg, or 20 mg/kg omadacycline delivered IP (OMC 5, OMC 10, or OMC 20, respectively), or linezolid delivered SQ (LZDsq) (n=3 per group). Symbols indicate the concentration of antibiotics in each mouse.

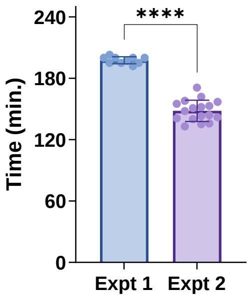

Supplementary Figure 2. Comparison of antibiotic administration time between Experiment 1 (Expt 1) and Experiment 2 (Expt 2). Bars represent mean, with SD. Symbols represent the time that treatment was administered post-MRSA infection. Significance was determined by unpaired t-test. ****p<0.0001

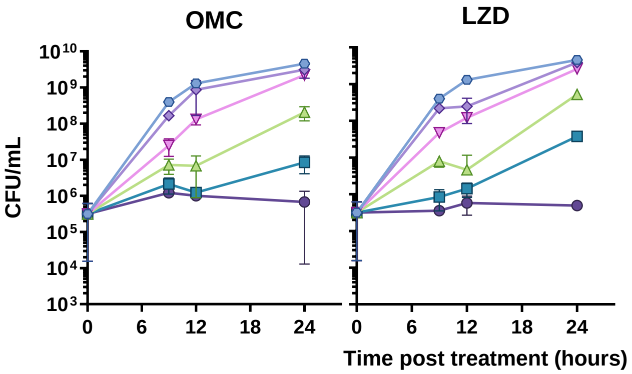

Supplementary Figure 3. Growth analysis of MRSA LAC strain treated with antibiotics. Growth was monitored over 24-hours with OMC (omadacycline), LZD (linezolid), NAF (nafcillin), or CEP (cephalothin). Symbols represent the time at which samples were taken to quantify bacteria (by CFU) and toxin production. Figure legends indicate a no treatment control (NT) or the fraction of the MIC. Data shown are from n = 3 done in triplicate. Error bars represent SEM.

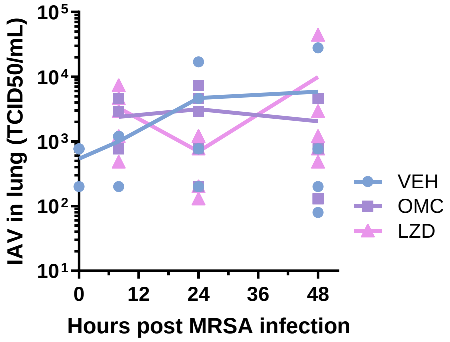

Supplementary Figure 4. Effects of omadacycline and linezolid on viral titer in IAV-MRSA infected mice. Mice (n = 5 per group per timepoint) were infected with 150 PFU IAV for 7-days and then infected with 4.0 x 10^8 MRSA for 8-, 24-, or 48-hours. IAV-MRSA co-infected mice were treated IP with 5 mg/kg omadacycline (OMC), PO with 120 mg/kg linezolid (LZD), or IP with vehicle (VEH). Lines represent the mean IAV titer in the right lung tissue. The TCID50 of IAV was determined by serial dilutions of the virus on Madin-Darby canine kidney (MDCK) cell monolayers. VEH, vehicle; OMC, omadacycline; LZD, linezolid.

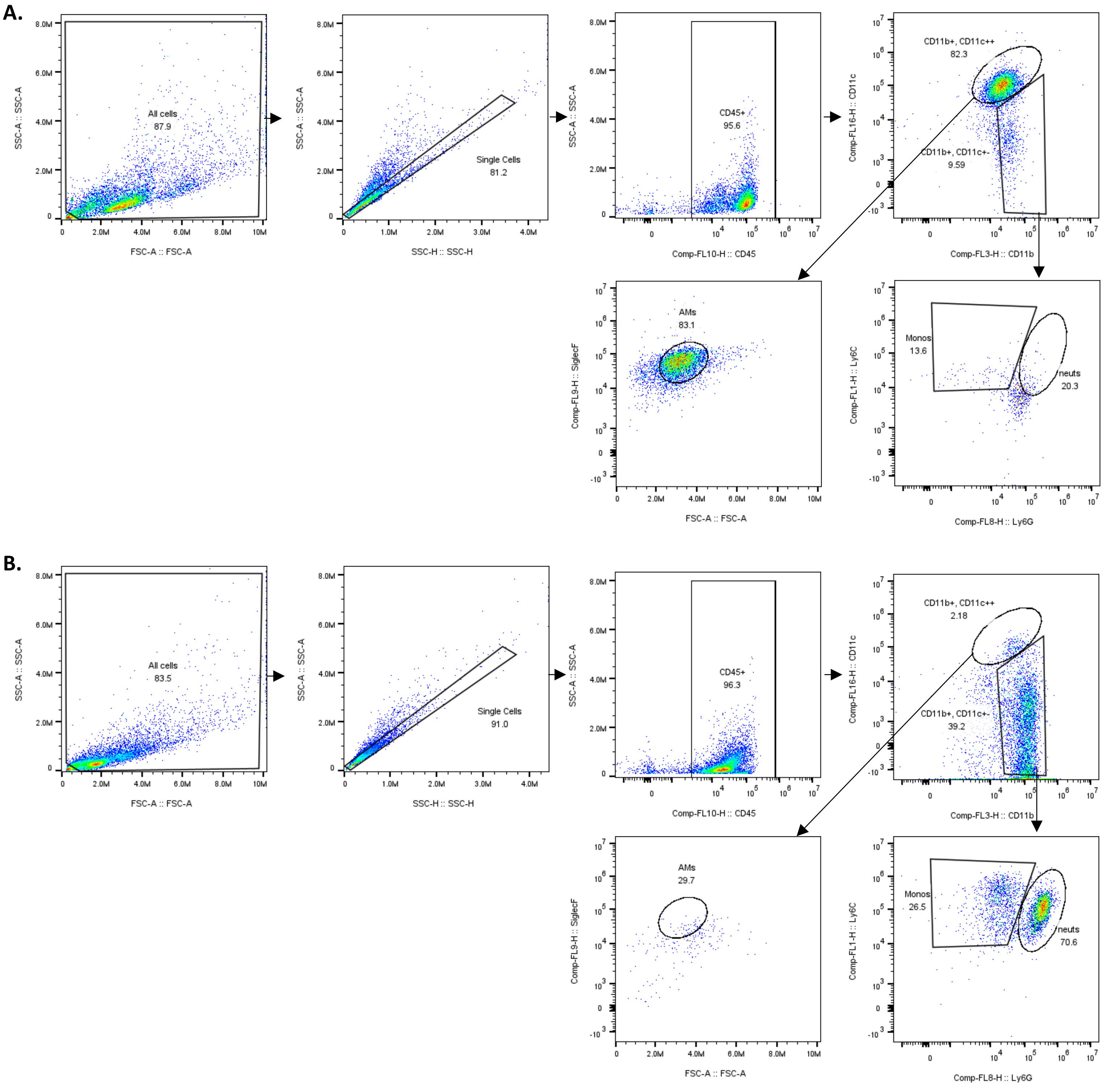

Supplementary Figure 5. Flow cytometry gating strategy for quantification of immune cells in BALF from A) healthy mice and B) IAV-MRSA co-infected mice treated with OMC at the 24-hour timepoint.

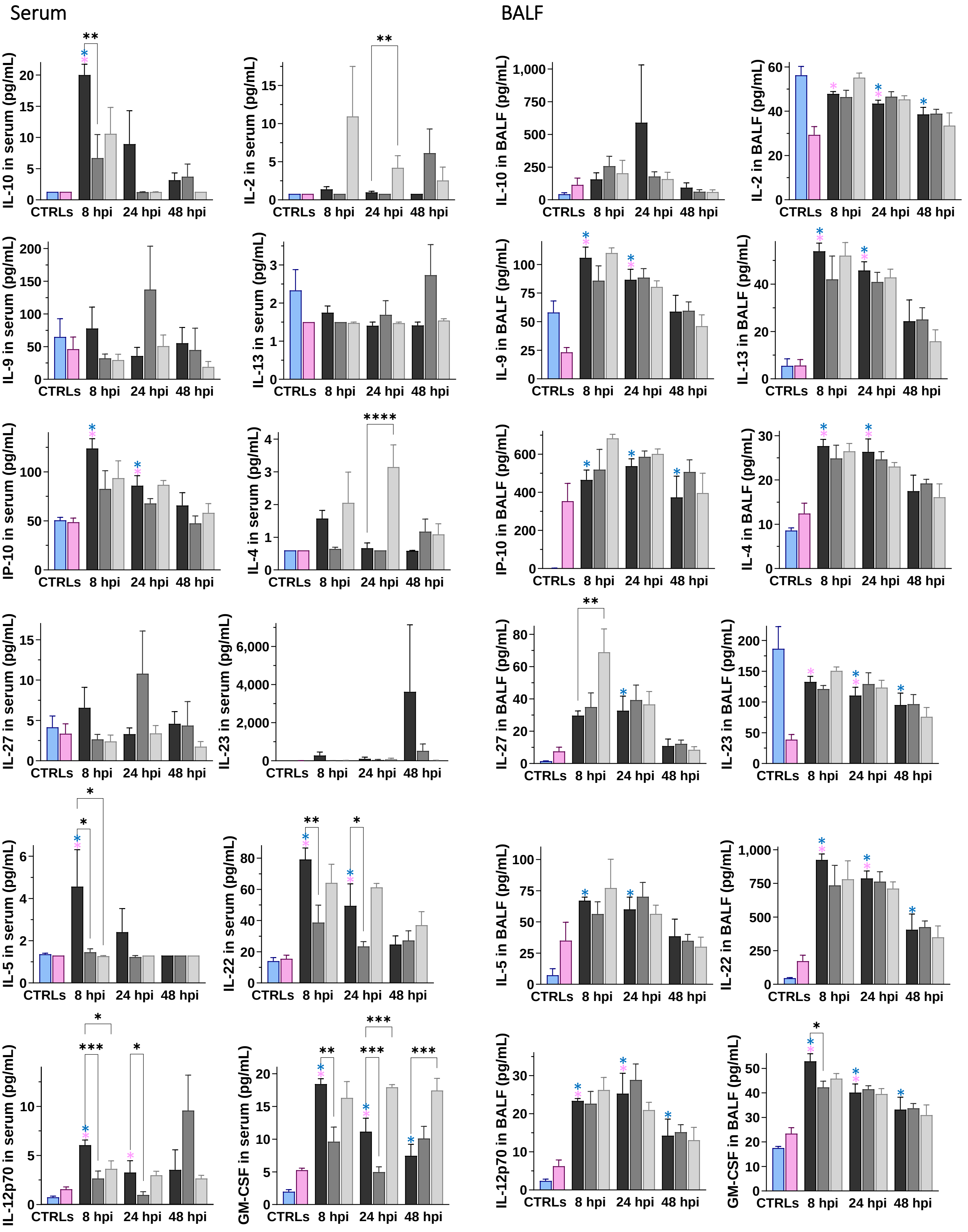
Serum
BALF
IL-10 in serum (pg/mL)
IL-2 in serum (pg/mL)
IL-10 in BALF (pg/mL)
IL-2 in BALF (pg/mL)
IL-9 in serum (pg/mL)
IL-13 in serum (pg/mL)
IL-9 in BALF (pg/mL)
IL-13 in BALF (pg/mL)
IP-10 in serum (pg/mL)
IL-4 in serum (pg/mL)
IP-10 in BALF (pg/mL)
IL-4 in BALF (pg/mL)
IL-27 in serum (pg/mL)
IL-23 in serum (pg/mL)
IL-27 in BALF (pg/mL)
IL-23 in BALF (pg/mL)
IL-5 in serum (pg/mL)
IL-22 in serum (pg/mL)
IL-5 in BALF (pg/mL)
IL-22 in BALF (pg/mL)
IL-12p70 in serum (pg/mL)
GM-CSF in serum (pg/mL)
IL-12p70 in BALF (pg/mL)
GM-CSF in BALF (pg/mL)
CTRLs
8 hpi
24 hpi
48 hpi

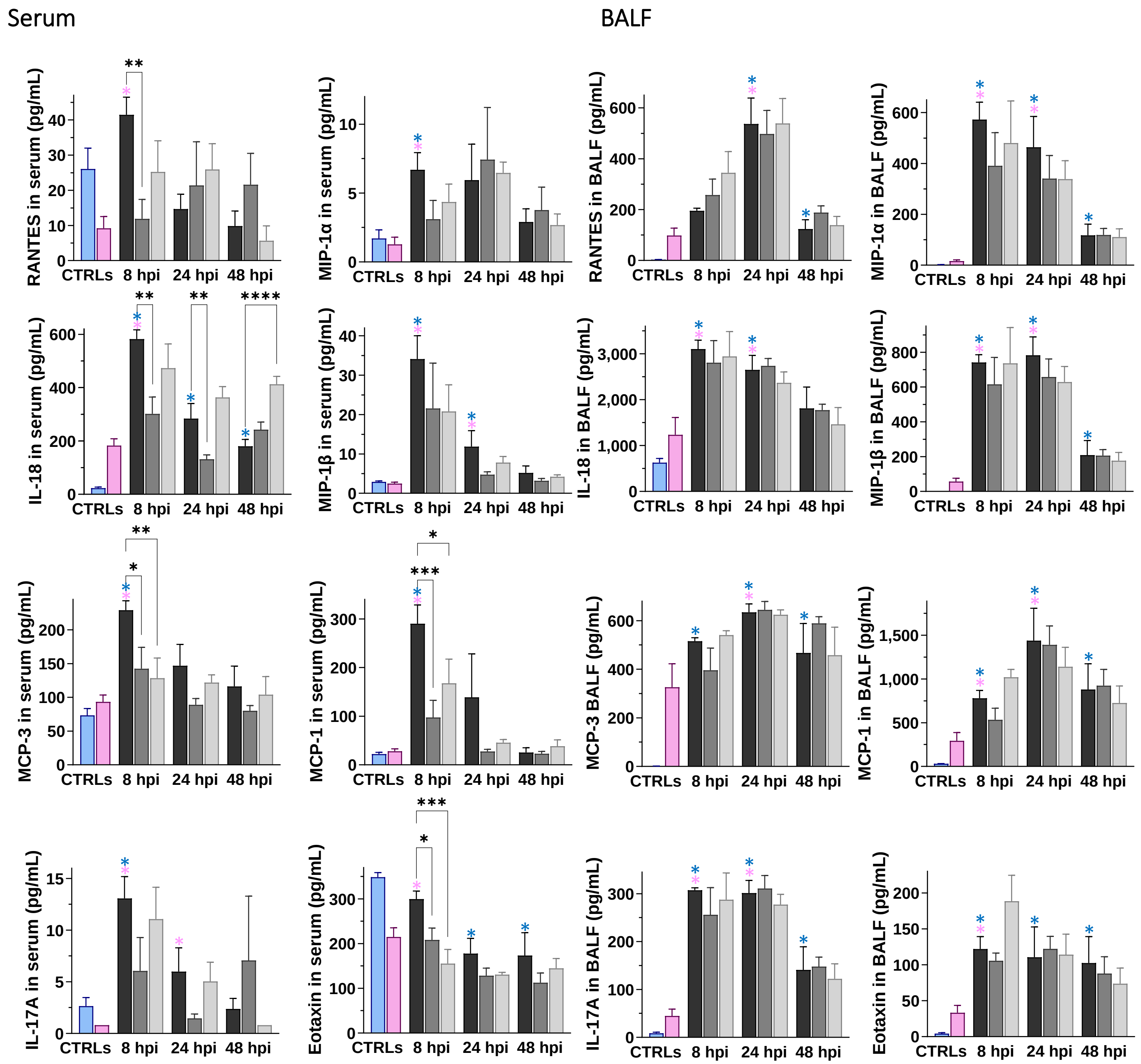

Supplementary Figure 6. Effects of omadacycline (OMC) and linezolid (LZD) on cytokine production. Graphs illustrate the average concentration of cytokines in serum and BALF for each treatment group at indicated times post-MRSA infection. Controls (CTRLs) include healthy (HEA) and IAV-only infected (IAV) mice. Symbols represent individual mice. Statistical significance was determined by one-way ANOVA and compared to vehicle (VEH) by Dunnett's multiple comparisons test post hoc, *p ≤0.05, **p≤0.01, ***p≤0.001, ****p≤0.0001. Bars represent the mean. Error bars represent SEM.

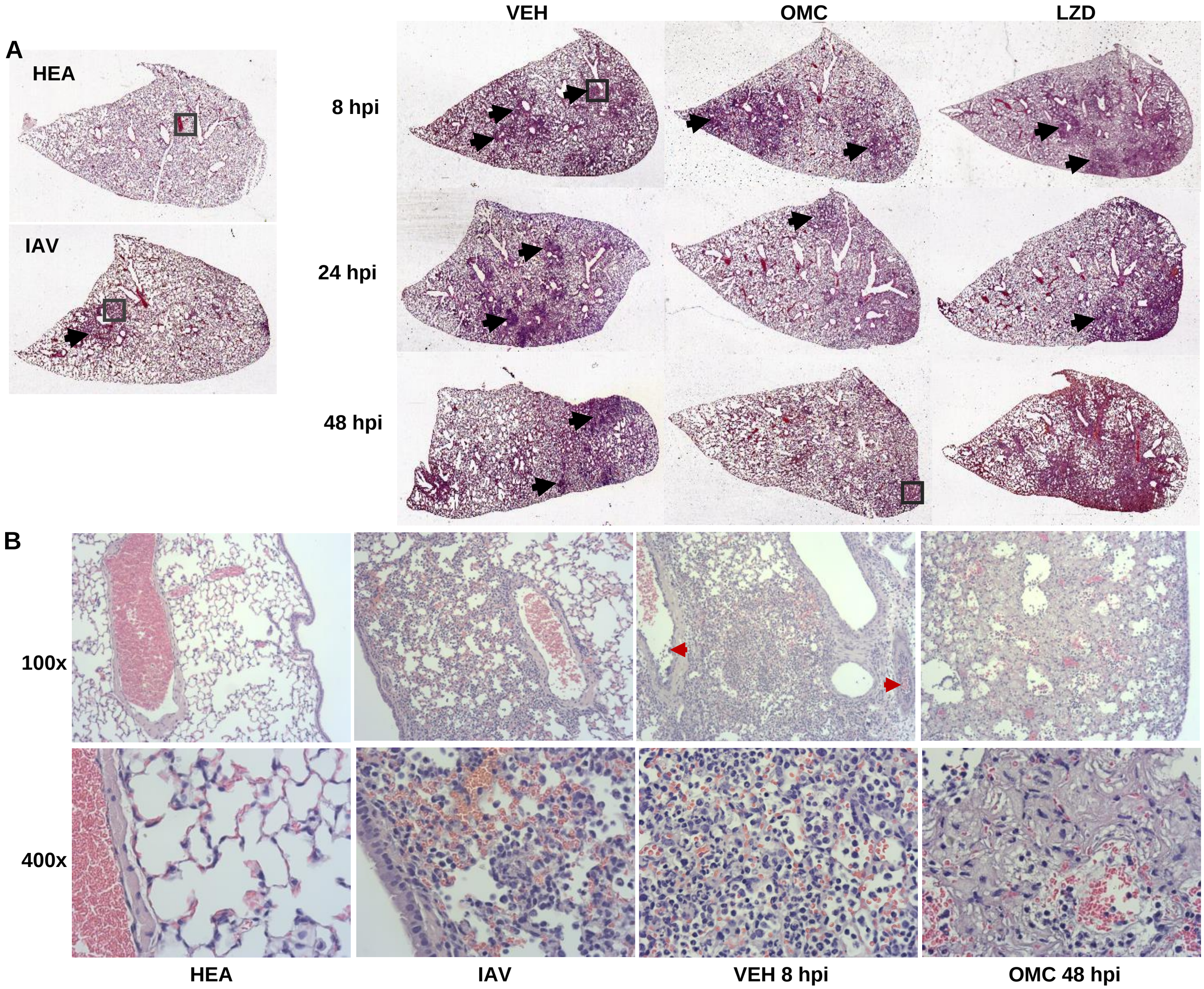

Supplementary Figure 7. IAV-MRSA co-infection histopathology. Hematoxylin and eosin-stained left lung tissues (representative of an n = 2). **A).** Slide scan images (20x). Black arrows point to examples of dense immune cell infiltrate. Boxes correlate with images in **B)**. **B).** 100x and 400x micrographs of the alveolar lumen and septa from lungs of healthy, IAV infected, and IAV-MRSA infected mice. Examples of vascular congestion (red arrows) are indicated.
